# Efforts Focused on Fatty Liver over Two Years (2023-2025): A Thematic Literature Review 

**DOI:** 10.30699/ijp.2025.2047720.3391

**Published:** 2025-08-15

**Authors:** Mahdi Abdorrashidi, Mohammad Hossein Peypar, Amirmohammad Tohidinia, Fatemeh Zali, Sobhan Eisazadeh, Mohammad Ali Abyazi, Mohammad Heiat

**Affiliations:** 1 Student Research Committee, Baqiyatallah University of Medical Sciences, Tehran, Iran; 2 Baqiyatallah Research Center for Gastroenterology and Liver Diseases (BRCGL), Clinical Sciences Institute, Baqiyatallah University of Medical Sciences, Tehran, Iran

**Keywords:** Artificial Intelligence, Fatty Liver Disease, Mobile Application, Metabolic Dysfunction-Associated Steatotic Liver Disease, Non-alcoholic Fatty Liver Disease

## Abstract

**Background & Objective:**

Nonalcoholic fatty liver disease, recently recognized as metabolic dysfunction-associated steatotic liver disease (MASLD), is a key factor in the development of chronic liver disease and the progression of liver fibrosis. It plays a significant role in increasing the risk of cirrhosis and hepatocellular carcinoma. Given the rapid developments in this field, keeping information up-to-date is essential to prevent misconceptions and ineffective decision-making. This study employs a scientometric approach to review scientific literature and analyze recent findings, offering a comprehensive overview of the current state of research in this field.

**Methods:**

In this approach, we explored data related to publication metrics, public perceptions, scientific conference, mobile apps, AI tools, and new medications. This was done using a set of keywords, including "Non-alcoholic fatty liver disease," "Metabolic dysfunction-associated steatotic liver disease," "Mobile application," and "Artificial intelligence."

**Results and Conclusion:**

This research highlights significant scientific advances in the field of MASLD, including major scientific meetings, highly cited publications, and the latest FDA-approved therapies. In addition, it examines emerging digital tools and public search frameworks, providing a structured picture of recent developments. These findings provide a comprehensive view of the dynamic MASLD research landscape and emphasize the roles of AI, mobile apps, and emerging therapies in its management.

## Introduction

Nonalcoholic fatty liver disease (NAFLD) is now one of the leading causes of chronic liver disease worldwide, with a spectrum ranging from simple steatosis to nonalcoholic steatohepatitis (NASH) and liver fibrosis and cirrhosis (1). The global prevalence of NAFLD has been rising at an alarming rate, closely associated with the increasing incidence of diabetes and obesity (2, 3). A recent meta-analysis on NAFLD estimated a global prevalence of 30.1% and observed a significant increase from 25.3% in 1990–2006 to 38.2% in 2016–2019. There were also regional differences, with the highest prevalence being in Latin America (44.4%) and in the Middle East and North Africa (36.5%) (4). Mathematical modeling studies on the global prevalence of NAFLD have reported that prevalence is predicted to be as high as 55.7% by 2040, with significant regional variations (5).

Lazarus et al. emphasize that national NAFLD guidelines are available in only 32 countries and that no country has a concerted public health response to address the disease (6). Global awareness, research funding, and policy initiatives will be needed to combat the rising NAFLD crisis. This emerging global health crisis warrants an urgent need to understand the various facets of NAFLD. 

Due to the heterogeneous nature of NAFLD and its association with metabolic risk factors, an expert panel replaced the term NAFLD with metabolic dysfunction-associated fatty liver disease (MAFLD) in 2020. The MAFLD terminology was considered stigmatizing because of the "fatty" term. Therefore, in 2023, it was replaced with "metabolic dysfunction-associated steatotic liver disease" (MASLD), which excluded the "fatty" term and reflected a more comprehensive understanding of the condition. This modification acknowledges that MASLD does not simply result from abstinence from alcohol but is the result of multiple complex interactions with cardiometabolic and environmental risk factors (7-9). In this study, we will use MASLD and NAFLD interchangeably, as most scientific literature still references the older terminology. 

A comprehensive understanding of the etiology, manifestations, and effects of MASLD is essential for its effective management. Enhancing our knowledge in these areas could significantly improve prevention, diagnosis, and treatment strategies. This study aimed to briefly define and describe each axis by reviewing scientific resources. The most important axes include hot topic papers, highly cited papers, scientific conferences, public mindset, mobile apps, artificial intelligence (AI), and new pharmaceutical products.

## Materials and methods

### Publication metrics

In the initial step, a time-restricted search (January 2023 to April 2025) was conducted using PubMed, Scopus, and Web of Science (WOS) to identify the number of published articles, the most active author, and the most cited article. The records were limited to English-language publications. However, to determine the most cited articles and active authors up to 2025, the search was conducted without any time restrictions.

The search strategy utilized specific keywords, including *Fatty Liver Disease*, *Fatty Liver*, *Nonalcoholic Fatty Liver Disease*, *NAFLD*, *Nonalcoholic Steatohepatitis*, *NASH*, *Alcoholic Steatohepatitis*, *Alcoholic Fatty Liver Disease*, *Metabolic-associated Fatty Liver Disease*, *Metabolic Dysfunction-associated Fatty Liver Disease*, *MAFLD*, *Metabolic Dysfunction-associated Steatotic Liver Disease*, *MASLD*, *Metabolic Dysfunction-associated Steatohepatitis,* and *MASH*. 

### Public Mindset

To assess the public mindset and analyze their search rate on the worldwide web from January 1, 2023, to April 12, 2025, a Google Trends analysis was conducted using the keywords *fatty liver disease*, *fatty liver*, *NAFLD,* and *NASH*.

### International scientific meetings

A comprehensive search was conducted across Google and pharmaceutical industry websites to identify scientific meetings related to fatty liver disease, documenting their most recent occurrences, locations, and related news.

### Health widgets

Randomized controlled trials (RCTs) examining the impact of mobile apps on patients with NAFLD or MASLD were recovered from 2023 to April 2025 through specific keywords such as *app*, *application*, *AI*, *Artificial intelligence,* *Nonalcoholic Steatohepatitis*, *NASH*, *NAFLD*, *MAFLD*, *Metabolic* *Dysfunction-associated Fatty Liver Disease* and *MAFLD* in PubMed, Scopus, and WOS databases. The search results were also screened for RCTs utilizing AI tools.

### Emerging therapeutic agents

Clinical trial records for recently developed drugs were reviewed from 2023 to April 2025. A search was conducted using the same keywords as those used for publication metrics, with RCT restrictions in PubMed, Scopus, and WOS.

## Results

### Publication metrics

The most-cited paper of 2023-2025, authored by M.E. Rinella et al., suggested using the term "MASLD" instead of "NAFLD" to better describe the underlying causes of the condition. This study has been cited approximately 1338 times (9).

The investigations revealed that the most cited study from the first record to April 2025 was conducted by Kleiner et al. (2005). Their study aimed to develop a scoring system for histological features in fatty liver and has been cited approximately 8419 times (10).

The most active researchers up to April 2025 and from 2023 to 2025 were Dr. Younossi and Dr. Loomba, respectively. Dr. Younossi, a well-respected hepatologist, has written 914 articles on this subject from the first record to April 2025 (11). Dr. Loomba, a globally known hepatologist, published 184 articles between 2023 and 2025 (12) (Table 1). 

**Table 1:** The most cited papers and active researchers.

Database	Total studies (2023 – 2025)	RCT studies (2023 – 2025)	Review studies (2023 – 2025)	The most cited study up to April 2025 (Citation n)	The most cited study 2023 – 2025 (Citation n)	Top Researcher up to April 2025 (Paper n)	Top Researcher 2023 – 2025 (Paper n)
PubMed (title/abstract)	19774	339	3700	-^a^	-^a^	-^a^	-^a^
Scopus (title/abstract/key)	32280	-^a^	4558	Design and validation of a histological scoring system for nonalcoholic fatty liver disease (8851) (10)	A multi-society Delphi consensus statement on new fatty liver disease nomenclature (1329) (9)	Loomba, Rohit (547)	Loomba, Rohit (184)
WOS (title)	27662	-^a^	3672	Design and validation of a histological scoring system for nonalcoholic fatty liver disease (8419) (10)	A multi-society Delphi consensus statement on new fatty liver disease nomenclature (1338) (9)	Younossi, Zobair M (914)	Noureddin, Mazen (182)

a: Unfilterable in the initial record.

These findings indicate that MASLD is the study trend for 2023-2025, with the most cited research focusing on its nomenclature and defining a term to describe such a situation. Replacing NAFLD with MASLD represents the most important and profound change in scientific definitions related to fatty liver, which has been accepted by specialists. It is expected that following this change, protocols and guidelines will be significantly revised.

*Public mindset*

**Fig 1:**
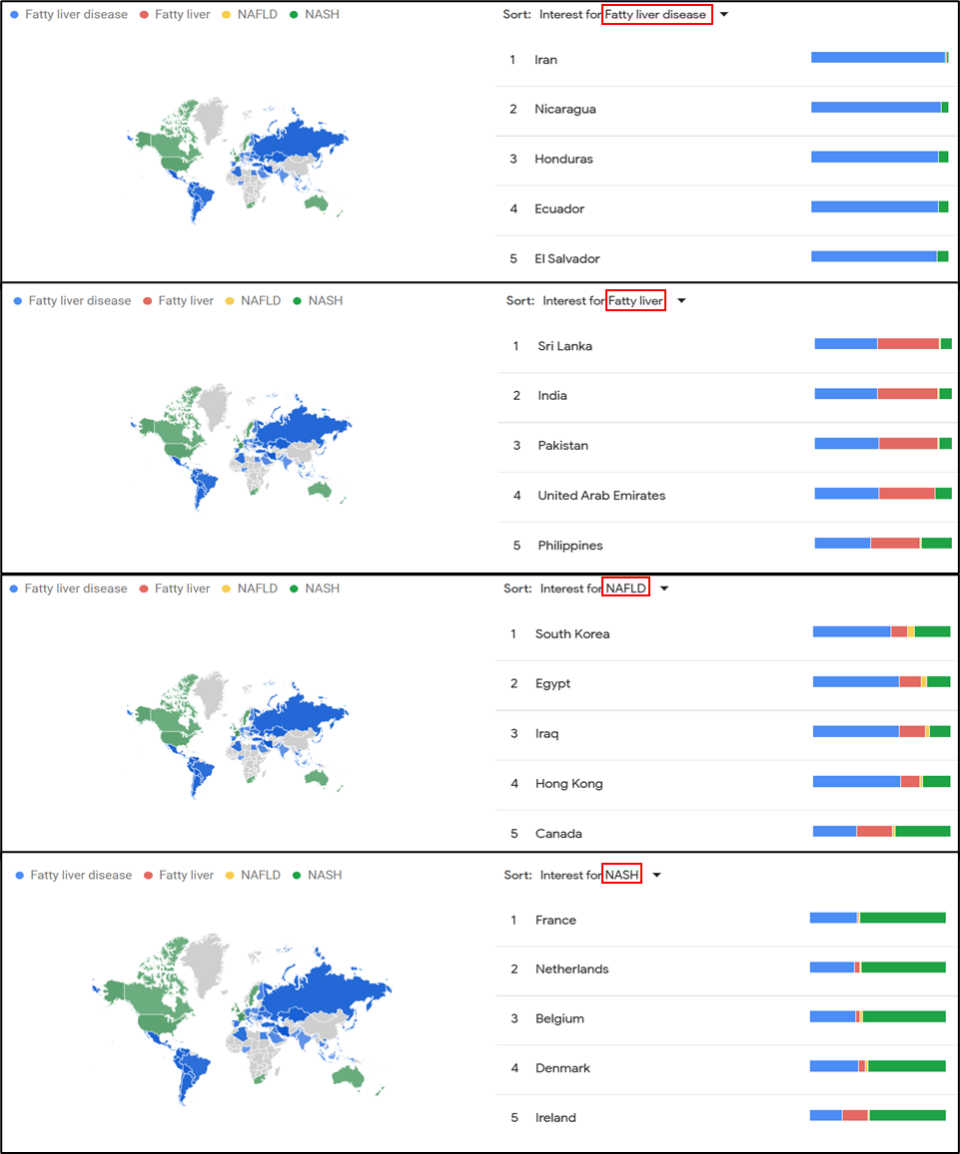
Regions with the highest search interest for each term. “Fatty liver disease” was most searched in Iran, “fatty liver” in Sri Lanka, “NAFLD” in South Korea, and “NASH” in France.

**Fig 2:**
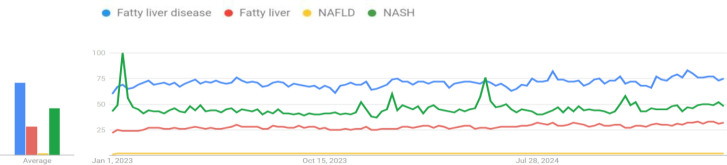
Search rank of each term in worldwide web search from 1/1/2023 to 4/12/2025. The most searched term during the first two weeks was “NASH” after which “fatty liver disease” became the most searched term.

The term "fatty liver disease" was the most searched keyword among others ("NASH," "fatty liver," and "NAFLD") (figure 1). The countries with the highest search volumes were Iran for "fatty liver disease," Sri Lanka for "NASH," South Korea for "fatty liver," and France for "NAFLD" (figure 2). Intriguingly, there is a difference in search methods among countries in different regions. So, Asian and South American countries favored the term "fatty liver disease," while North American countries preferred "NASH." This variation in search patterns may indicate differences in disease prevalence or the way these conditions are addressed and expressed in different regions. 

### International scientific conferences

Several international scientific conferences are dedicated to liver disease, with a particular focus on MASLD. These events bring together researchers, healthcare professionals, and specialists from around the world to share their latest advancements in the field. The most important international events in 2023-2025 were summarized in Table 2. 

**Table 2:** International events.

**Event**	**last held time**	**location**	**Hot topics discussed in MASLD/MASH**	**Ref**
AASLD^a^	Nov / 10-14 / 2023	Boston, Massachusetts	23% Grow of the MASLD and MASH^b^ in the US through 2050.	(13)
			Reduction in inflammation markers and liver fat content with Low-Dose Aspirin.	
			liver measures can be improved with Weight-Loss drugs.	
	Nov / 15-19 / 2024	San Diego, California	Bariatric surgery better than obesity drugs for some patients with MASLD.	(14)
			AI tool identifies undiagnosed early-stage MASLD.	
			Watershed moment’: semaglutide shown to be effective in MASH.	
			Some antihypertensives linked to HCC^c^ risk in patients with MASLD and cirrhosis.	
EASL^d^	June / 21-24 / 2023	Vienna, Austria	Pegozafermin for the treatment of NASH patients with F2/F3 fibrosis.	(15)
			Effect of pemvidutide, a GLP-1^e^/glucagon dual receptor agonist, on MASLD.	
			Effectiveness of time-restricted intermittent fasting in patients with NAFLD.	
			Endoscopic sleeve gastroplasty plus lifestyle intervention in patients with MASH.	
	June / 5-8 / 2024	Milan, Italy	Tirzepatide can improve the MASH resolution and fibrosis.	(16)
			Dramatic' phase 2 results for survodutide in MASH, fibrosis.	
The International NASH Day	June / 13 / 2024	Washington, DC^f^	Machine-based approach algorithm can be used for rapidly identifying patients with MASH, with high accuracy.	(17)
			Fatty liver disease is a common condition that can damage liver but it is reversible and preventable if detect early.	

^a.^AASLD: American Association for the Study of Liver Diseases, ^b^. MASH: Metabolic Dysfunction-associated Steatohepatitis, ^c^. HCC: Hepatocellular carcinoma, ^d^. EASL: European Association for the Study of the Liver, ^e^. GLP-1: glucagon-like peptide, ^f^. DC: the District of Columbia.

### Health widget

#### Mobile apps

With advancements in technology, data, and the widespread use of smartphones, it is possible to develop mobile applications supported by a team of medical professionals. These applications, like a nutrition specialist or a certified personal trainer, can recommend personalized nutritional and exercise programs based on an individual's lifestyle and limitations. 

Weight loss is considered the most effective treatment for overweight or obese patients with NAFLD (18). Numerous available mobile applications have the potential to assist patients in managing and treating NAFLD by monitoring dietary habits and facilitating weight loss. Four clinical trial studies on mobile applications were retrieved, demonstrating reliable outcomes in improving MASLD. The clinical trial for the "SMART-Liver" application had the largest sample size among the four applications and showed a significant effect on weight, body mass index (BMI), liver fat score, quality of life, and self-regulation. However, the most downloaded application with the highest score in the Google Play Store is the Noom Weight application. While the Noom Weight application showed significant improvements in body weight and BMI, it was not successful in addressing critical clinical features for NAFLD, such as the NAFLD Fibrosis Score (NFS) and FIB-4 Index (FIB-4) (19, 20).

Furthermore, a recent study investigated the use of the Better Therapeutics app, a form of digital Cognitive Behavioral Therapy (CBT), for patients with MASLD or Metabolic Dysfunction-associated Steatohepatitis (MASH). Over a 90-day intervention, participants experienced significant improvements in LFC (liver fat content), AL, and an average body weight loss of nearly 2.9%, all without any device-related adverse events. Based on these promising clinical results, the app was granted FDA breakthrough status in February 2024 (Table 3) (21, 22).

**Table 3:** Characteristics and outcomes of clinical trials evaluated the effects of mobile apps in MASLD.

APP	Author	Study duration	Participants (n)	Used app (n)	Comparator arm (n)	Strategy used for treatment	Effect	Ref
SMART-Liver	Kwon et al.	26-Week	Patients with NAFLD (111)	SMART-Liver (51)	Standard care (60)	Self-tracking, education, coaching and SMS text messages	Weight reduction (P<0.001) BMI Reduction (P<0.001) liver fat score reduction (P=0.01) AST reduction (P=0.03) ALT reduction (P=0.002) ɣ-GT reduction (P=0.04) Self-regulation improvement (P<0.001) Depression improvement (P=0.003) Fatigue improvement (P=0.005) Quality of life scores improvement (P<0.001)	(23)
Noom Weight	Jonathan et al.	16-Week	Patients with NASH and a smartphone (40)	Noom Weight (20)	Standard care (20)	Self- tracking, feedback, education and motivation	Weight reduction (P = 0.008) BMI reduction (P = 0.037) AST reduction (P = 0.310) ALT reduction (P = 0.547) ALPs reduction (P = 0.838) NFS improvement (P = 0.444) FIB-4 improvement (P = 0.733)	(24)
NASH app	Sato et al.	48-Week	Patients with NASH or NAFLD (20)	NASH app (20)	_	Advice, education, and counseling sessions	NAS^a^ reduction (P < 0.001) Weight reduction (P < 0.001) AST reduction (P = 0.001) ALT reduction (P < 0.001) ɣ-GT reduction (P = 0.02) ALPs reduction (P < 0.001) TG reduction (P = 0.01) Improvement in parameters related to insulin resistance (P > 0.05)	(25)
Better Therapeutics app	Alkhouri et al.	90-Day	Patients with MASLD or MASH (22)	Better Therapeutics app (22)	-	Digital therapeutic delivery, CBT, standardized lessons, personalized goal-setting, and self-monitoring	Weight reduction (P = 0.008) ALT reduction (P = 0.007) FIB-4 improvement (P = 0.045)	(21)

^a^. NAS: nonalcoholic fatty liver disease activity score.

These studies have shown that mobile applications can significantly decrease weight and BMI and are effective in reducing liver enzymes (AST, ALT, ɣ-GT), except the "*Noom Weight*" application. However, none of the applications showed a significant effect on NFS, FIB-4, and insulin resistance. This may be because NFS and FIB-4 indices include variables such as age, platelet count, and albumin level, which are typically not affected by the short-term lifestyle changes that these applications support. In addition, the lack of effect of the *Noom Weight* app on liver enzymes may be related to its short-term follow-up period, suggesting that long-term studies are needed to more accurately assess its potential benefits. Overall, although mobile apps are promising tools for weight management and improving some biochemical markers, their efficacy in improving advanced liver fibrosis remains limited. As a result, more comprehensive research with optimized designs is needed to assess the long-term effects of these programs.

### Artificial Intelligence (AI) 

AI can revolutionize healthcare by enhancing patient care, improving the experience of caregivers, and reducing expenses. AI is playing an expanding role in evaluating and diagnosing the risk of fatty liver disease. In this context, risk factors are frequently utilized for modeling, and pathological images are applied in image processing. AI systems possess the ability to predict problems and adjust processes accordingly. It acquires knowledge from extensive datasets to aid professionals in the identification and management of medical conditions (26). AI can be used in the management of NAFLD by identifying the risk factors, diagnosing severity, and drug development (27). Two studies were retrieved that assessed AI potentials in diagnosing MASLD. However, studies have shown that there is only a fair to moderate agreement between human and AI diagnoses (Table 4).

**Table 4:** AI role in MASLD.

AI	Author	Type of study	Study duration	Participants (n)	Comparator arm	Recruitment	Outcome	Ref
ANN^a^	Liu C et al	Cross-sectional	-	American people (6,613)	-	Predicting the risk of NAFLD	NAFLD was observed when the estimated risk of its occurrence exceeded the calculated threshold (0.388) using the established ANN model.	(28)
Path AI’s NASH ML^b^	Ratziu V et al	Randomized, double-blind, placebo-controlled trial	72-week	NASH patient with confirmed biopsies (251) and fibrosis stage F1-F3	Evaluated histological features of NASH by pathologists	Evaluating histological features of NASH	The agreement between AI and pathologists ranged from 0.28 to 0.62 weighted kappa statistics across histological characteristics. Steatosis (0.46–0.62) had the highest agreement, while fibrosis, lobular inflammation, and hepatocyte ballooning had lower.	(29)

^a^. ANN: artificial neural network, ^b^. ML: machine-learning.

This suggests that AI has great potential, yet it’s still not ready to fully replace human expertise. Several fundamental challenges remain to be overcome. These include the need to increase the accuracy of algorithms, ensure transparency of their performance, and accurately assess effectiveness in diverse patient populations. In addition, AI tools need to act as decision-making assistant for physicians, not a replacement for them, especially given the risks of misdiagnosis or misclassification that may arise from biases or limitations in the training data.

### Emerging therapeutic agents

The management of MASLD primarily focuses on lifestyle changes, particularly in nutrition and exercise routines, with the main objective of achieving weight loss (18). However, several biochemical mechanisms and therapeutic agents have been proposed as potential treatments for MASLD.

Table 5 provides a summary of clinical trials evaluating the efficacy of emerging therapeutic agents with various mechanisms of action ranging from hormone receptor agonists (e.g., fibroblast growth factor-21(FGF-21) analogues such as Pegbelfermin) to enzyme inhibitors (e.g., ketohexokinase (KHK) inhibitor PF-06835919) for MASLD treatment. *Resmetirom* is currently the only drug approved by the FDA. The clinical trial for *Resmetirom* trial included three double-blind groups (100 mg, 80 mg, and a placebo) along with an open-label group receiving 100 mg. The results showed that *Resmetirom* was both safe and well-tolerated, leading to significant improvements in liver enzymes, lipid profiles, LFC, and liver stiffness (30). 

## Conclusion

Today's world has various complications and is sometimes harmful to human health. Drowning and disappearing in these intertwined problems is possible for many aspects of human beings. Perhaps one of the solutions to overcome this complex and extensive diversity is to acquire scientific knowledge. This issue becomes more important for physicians and researchers. The abundance on findings and information of scientific events and their rapid increase can easily confuse researchers and physicians. One of the tools with high potential to deal with this issue is the scientometric tool, which can be used to categorize and configure data. The output of this tool can identify the most important, the most abundant, the best, the worst, the most recent, and the most useful ones for the researchers and provide the basis for making a correct decision. As in this study, by using this technique, we succeeded in identifying the most important scientific conferences (*EASL*, *AASLD*), the most cited scientific publications (*Design and validation of a histological scoring system for nonalcoholic fatty liver disease and a multi-society Delphi consensus statement on new fatty liver disease nomenclature*), the newest effective drug on MASLD that has been approved by the FDA (*Resmetirom*). We also identified the most useful tools (*AI and lifestyle mobile apps*) and provided a relatively comprehensive picture of the latest scientific events. Conducting such research in regular periods can effectively provide comprehensive decision-making concepts for researchers and draw a clear and attractive road map.

**Table 5:** Characteristics and outcomes of clinical trials investigated the effects of emerging therapeutic agents for MASLD (Significantly increase (↑) significantly decrease (↓)).

**Drug**	**Type of RCT**	**Intervention group(n)**		**Comparator arm (n)**		**Duration**	**Mechanism of action**	**Outcome**	**Ref**
Resmetirom	Randomized, double-blinded, placebo-controlled trial	Resmetirom 100mg daily (325)			Placebo (320)	52-Week	THR-β5^a^ agonist	LFC↓, liver stiffness↓, TG↓, ALT↓, AST↓. The most TAEs^b^ were mild to moderate gastrointestinal events (nausea, vomiting, diarrhea).	(30)
		Resmetirom 80mg daily (327)							
		Resmetirom 100mg open-label daily (171)							
Efinopegdutide	Randomized, open-label active-comparator-controlled, parallel-group trial	Efinopegdutide 10mg once weekly (72)			Semaglutid 1mg once weekly (73)	32-Week	GLP-1/glucagon dual receptor agonist	LFC↓. Nausea and vomiting were the most frequent TAEs across the study; however, the efinopegdutide showed a higher overall incidence of TAEs.	(31)
Vonafexor	Randomized, double-blinded, placebo-controlled trial	Part A	Vonafexor 400 mg daily (17)		Placebo (7)	14-Week	FXR^c^ agonist	LFC↓, bodyweight↓. The most TAEs were mild to moderate pruritus.	(32)
		Part B	Vonafexor 100mg daily (31)		Placebo (32)			LFC↓, bodyweight↓. The most TAEs were mild to moderate pruritus.	
			Vonafexor 200mg daily (33)						
Genotropin	Randomized, double-blinded, placebo-controlled trial	Genotropin 0.5 ± 0.2 mg daily (27)			Placebo (26)	6-Month	GHR^d^ agonist	LFC↓, visceral fat↓, ALT↓, IGF-1^e^↑. No serious TAEs or major safety concerns were reported during the study. The most common TAEs observed were edema, injection site discomfort or bruising, joint pain or stiffness and myalgias.	(33)
ZSP1601	Randomized, double-blinded, placebo-controlled trial	ZSP1601 50mg once daily (9)			Placebo (3)	4-Week	Pan-PDE^f^ inhibitor	ALT↓, AST↓. No serious TAEs or major safety concerns were reported during the study.	(34)
		ZSP1601 50mg twice daily (9)			Placebo (3)				
		ZSP1601 100mg twice daily (9)			Placebo (3)				
PF-06835919	Randomized, double-blinded, placebo-controlled trial	PF-06835919 150mg daily (55)			Placebo (54)	16-Week	KHK inhibitor	LFC↓ in PF-06835919 300mg group. The incidence of TAEs was similar across groups, with the most frequently reported TAEs being diarrhea, headache, and abdominal pain.	(35)
		PF-06835919 300mg daily (55)							
PXL065	Randomized, double-blinded, placebo-controlled trial	PXL065 7.5 mg daily (25)			Placebo (30)	36-Week	MPC^g^/ACSL4^h^ inhibitor	LFC↓, NFS↓, insulin sensitivity↑. While the overall incidence of TAEs was similar across groups, the PXL065 22.5 mg group showed a slightly higher frequency of headache, nausea, and abdominal pain.	(36)
		PXL065 15mg daily (32)							
		PXL065 22.5mg daily (30)							
Aldafermin	Randomized, double-blinded, placebo-controlled trial	Aldafermin 0.3mg daily (7)			Placebo (56)	48-Week	FGF-19^i^ analogue	ALT↓, AST↓, liver stiffness↓. The mild and moderate gastrointestinal events were the most frequent TAEs across the study; however, the aldafermin 3mg showed a higher incidence of diarrhea.	(37)
		Aldafermin 1mg daily (42)							
		Aldafermin 3mg daily (55)							
Nicotinamide Riboside and Pterostilbene (NRPT)	Randomized, double-blinded, placebo-controlled trial	NRPT 1× (250 mg NR and 50 mg PT) daily (41) NRPT 2× (500 mg NR and 100 mg PT) daily (37)			Placebo (33)	6-Month	Oxidative metabolism inducer	Fatty liver index ↓, ALT↓, AST↓, insulin sensitivity↑. The incidence of TAEs was similar across groups, with the most frequently reported TAEs being gastrointestinal events.	(38)
Misoprostol	Randomized, double-blinded, placebo-controlled trial	Misoprostol 0.2 mg thrice daily (25)			Placebo (25)	2-Month	Anti-inflammatory modulator	ALT↓, AST↓. The incidence of TAEs was similar across groups, with the most frequently reported TAEs being diarrhea.	(39)
Dapagliflozin	Randomized, open-label, controlled trial	Dapagliflozin 10 mg daily + metformin (40)			Non-SGLT2^j^ inhibitor antidiabetic + metformin (38)	24-Week	SGLT2 inhibitor	LFC↓, PFC^k^↓, TNF-α↓, IL-6↓, ALT↓, bodyweight↓. No significant adverse events were reported in this study.	(40)
Ertugliflozin	Randomized, double-blinded, placebo-controlled trial	Ertugliflozin 15 mg daily (60)			Placebo (60)	24-Week	SGLT2 inhibitor	LFC↓, body weight↓, AST↓, ALT↓, insulin sensitivity↑. No significant adverse events were reported in this study.	(41)
					Pioglitazone 30 mg daily (60)				
Pegbelfermin	Randomized, double-blinded, placebo-controlled trial	Pegbelfermin 10 mg once weekly (39)			Placebo (39)	48-Week	FGF-21 analogue	Decreases in NAS, liver stiffness, steatosis, AST, and ALT were observed, but none of these improvements showed statistical significance. The incidence of TAEs was highest in the pegbelfermin 20 mg group, with the most frequently reported TAEs being diarrhea and nausea.	(42)
		Pegbelfermin 20 mg once weekly (37)							
		Pegbelfermin 40 mg once weekly (39)							
Efruxifermin	Randomized, double-blinded, placebo-controlled trial	Efruxifermin 28 mg daily (42)			Placebo (43)	96-Week	FGF-21 analogue	LFC↓, ALT↓, AST↓, TG↓, LDL↓, HDL↑, insulin sensitivity↑. The most frequent TAEs were mild to moderate diarrhea and nausea.	(43)
		Efruxifermin 50 mg daily (43)							
Ipragliflozin	Randomized, open-label, controlled trial	Ipragliflozin 50 mg daily (77)			Sitagliptin 50 mg daily (83)	6-Month	SGLT2 inhibitor	Body weight↓, AST↓, ALT↓, uric acid↓. The frequency of TAEs was higher in ipragliflozin group. Additionally, skin disease and elevated blood ketones were reported only in the ipragliflozin group.	(44)
Rosuvastatin	Randomized, open-label trial	Rosuvastatin 10 mg daily (16)			Negative control group (16)	52-Week	HMG-CoA^l^ inhibitor	LFC↓, LDL↓, TG↓, HDL↑. No significant adverse events were reported in this study.	(45)
Semaglutide	Randomized, double-blinded, placebo-controlled trial	Semaglutide 2.4 mg once weekly (47)			Placebo (24)	48-Week	GLP-1 agonist	LFC↓, body weight↓, ALT↓, TG↓. The difference in AST and NAS improvement was not statistically significant. The frequency of TAEs was similar between two groups. The most frequent TAEs were mild to moderate nausea, diarrhea and vomiting.	(46)
Cotadutide	Randomized, double-blinded, placebo-controlled trial	Cotadutide 0.3 mg daily (25)			Placebo (24)	19-Week	GLP-1/glucagon dual receptor agonist	LFC↓, body weight↓, ALT↓, AST↓. The most common TAEs were nausea, vomiting, decreased appetite, diarrhea, and headache, with a higher incidence observed in the cotadutide 0.6 mg group.	(47)
		Cotadutide 0.6 mg daily (25)							
Pemvidutide	Randomized, double-blinded, placebo-controlled trial	Pemvidutide 1.2 mg once weekly (23)			Placebo (24)	12-Week	GLP-1/glucagon dual receptor agonist	LFC↓, body weight↓, ALT↓. Maximal responses were observed at the 1.8 mg dose. TAEs were mild gastrointestinal events (nausea, diarrhea, vomiting, constipation) which resolved without treatment.	(48)
		Pemvidutide 1.8 mg once weekly (23)							
		Pemvidutide 2.4 mg once weekly (24)							
Retatrutide	Randomized, double-blinded, placebo-controlled trial	Retatrutide 1 mg once weekly (20)			Placebo (19)	48-Week	GLP-1/ Glucagon/GIP triple receptor agonist	LFC↓, body weight↓, abdominal fat↓, lipid metabolism↑, insulin sensitivity↑. With the reduction in LFC becoming more significant at higher drug doses. TAEs were mild to moderate gastrointestinal events (nausea, diarrhea, vomiting, constipation, change in bowel habits) with higher frequency in the 8 mg and 12 mg dose groups.	(49)
		Retatrutide 4 mg once weekly (19)							
		Retatrutide 8 mg once weekly (22)							
		Retatrutide 12 mg once weekly (18)							
Tirzepatide	Randomized, double-blinded, placebo-controlled trial	Tirzepatide 5 mg once weekly (47)			Placebo (48)	52-Week	GLP-1/GIP^m^ dual agonist	LFC↓, body weight↓, ALT↓, AST↓. The most TAEs were mild to moderate gastrointestinal events (nausea, diarrhea, decreased appetite, constipation).	(50)
		Tirzepatide 10 mg once weekly (47)							
		Tirzepatide 15 mg once weekly (48)							

^a^. THR-β5: Thyroid hormone receptor beta-5, ^b^. TAEs: Treatment adverse events, ^c^. FXR: Farnesoid X receptor, ^d^. GHR: Growth hormone receptor, ^e^. IGF-1: Insulin-like growth factor-1, ^f^. PDE: Phosphodiesterase, ^g^. MPC: Mitochondrial pyruvate carrier, ^h^. ACSL4: Acyl CoA synthetase 4, ^i^. FGF-19: Fibroblast growth factor-19, ^j^. SGLT2: Sodium-glucose co-transporter 2, ^k^. PFC: Pancreatic fat content, ^l^. HMG-CoA: Hydroxymethylglutaryl-CoA, ^m^. GIP: Gastric inhibitory polypeptide,.

## Notes on Contributors

M.A. conducted the literature search, selected the studies, and prepared the manuscript. M.P. conducted the literature search and edited the manuscript. A.T. prepared the manuscript and extracted data. M.A.A. wrote and edited the manuscript. F.Z. edited the manuscript and extracted data. S.E. was responsible for study selection and data extraction. M.H. evaluated data and conceptualized the manuscript. All authors read the article in full and approved it.

## Data Availability

There is no additional data separate from available in cited references.
